# Prevalence, Associated Factors, and Evaluation of Clinical and Biochemical Indices of Gestational Trophoblastic Tumors in Patients Referring to Imam Reza Hospital, Kermanshah: A Five‐Year Analytical Study (2017–2022)

**DOI:** 10.1002/hsr2.72739

**Published:** 2026-06-30

**Authors:** Nasrin Mansouri, Ali Azizi, Samira Sajadi Asl, Zahra Javanbakht, Marzieh Bagherinia

**Affiliations:** ^1^ Department of Obstetrics and Gynecology, Clinical Research Development Center, Imam Reza Hospital Kermanshah University of Medical Sciences Kermanshah Iran; ^2^ Department of Community and Family Medicine School of Medicine, Kermanshah University of Medical Sciences Kermanshah Iran; ^3^ Student Research Committee, School of Medicine Kermanshah University of Medical Sciences Kermanshah Iran; ^4^ PhD of Reproductive Health, Clinical Research Development Center, Motazedi Hospital Kermanshah University of Medical Sciences Kermanshah Iran

**Keywords:** gestational trophoblastic disease, hydatidiform mole, Iran, neoplastic, pregnancy complications

## Abstract

**Background and Aims:**

Gestational trophoblastic neoplasia (GTN) encompasses a spectrum of pregnancy‐related disorders and poses significant clinical challenges. Understanding its prevalence, clinical characteristics, and associated risk factors is essential for timely diagnosis and management. This study aimed to evaluate the prevalence, clinical and biochemical features, and associated factors of GTN patients admitted to a tertiary referral center in northwestern Iran.

**Materials and Methods:**

A cross‐sectional, descriptive–analytical study was conducted on all women diagnosed with GTN at Imam Reza Hospital, Kermanshah, between 2017 and 2022. Data were collected retrospectively from both electronic and paper‐based medical records using a standardized checklist. Variables included demographics, obstetric and medical history, clinical signs, biochemical markers (β‐hCG), treatment modalities, and metastatic status. Descriptive statistics were analyzed using SPSS v26.

**Results:**

Among 30,133 deliveries during the study period, 382 GTN cases were identified, yielding a prevalence of 1.27%. Complete mole was the most frequent subtype (56.8%), followed by partial mole (37.7%). Vaginal bleeding (85.6%) and uterine enlargement (74.6%) were the most common clinical presentations. Blood group A was the most prevalent (47.4%), and 15.4% of patients had a history of consanguineous marriage. Uterine evacuation was the primary treatment (85.3%), with chemotherapy and hysterectomy used as indicated. Metastatic disease was observed in 7.6% of patients, predominantly affecting the lungs.

**Conclusion:**

GTN represents a notable health concern in this region, with complete mole being the most common form. Age, educational level, blood group, and prior molar pregnancy were identified as relevant factors. Early recognition based on clinical and biochemical markers, alongside structured follow‐up, is crucial to optimizing outcomes. These findings underscore the importance of awareness, education, and comprehensive care strategies for high‐risk populations.

## 1 Introduction

Gestational Trophoblastic Disease (GTD) refers to a specific spectrum of proliferative disorders of trophoblasts associated with pregnancy and originating from the placental epithelium [1]. From a classification perspective, GTD is divided into two main groups: molar diseases (hydatidiform mole) and other malignant trophoblastic neoplasms. These types differ fundamentally in histopathology and clinical course, and given their neoplastic nature, they can lead to life‐threatening consequences for the mother [2, 3]. Molar pregnancy is generally considered a non‐invasive form of GTD; however, these lesions have premalignant potential and, if left untreated, may progress to invasive forms [4]. Non‐molar or malignant forms of GTD are known as Gestational Trophoblastic Neoplasia (GTN), which includes invasive mole, choriocarcinoma, epithelioid trophoblastic tumor (ETT), and placental site trophoblastic tumor (PSTT). These lesions can appear weeks or even years after either a molar or non‐molar pregnancy and, if not diagnosed and treated promptly, are associated with high rates of metastasis and mortality [5]. Among them, choriocarcinoma is a rare but highly aggressive type of GTN, often arising in women following molar pregnancy, term pregnancy, or miscarriage [6, 7].

The prevalence of these diseases varies worldwide and is reported to be higher in Asian and developing countries than in developed nations. According to estimates, the incidence of hydatidiform mole is approximately 1 to 2 cases per 1000 pregnancies [8]. Multiple factors contribute to the occurrence of GTD, including maternal age under 20 or over 35 years, previous history of hydatidiform mole, multiple pregnancies, dietary habits, and Rh‐negative blood group [9, 10, 11]. Beyond biological and obstetric risk factors, growing evidence suggests that social and behavioral determinants of health may also influence women's reproductive outcomes and access to timely healthcare services. Socioeconomic status, educational attainment, social support, lifestyle behaviors, and health‐promoting practices during pregnancy have been shown to affect maternal well‐being and reproductive health indicators [12, 13].

Clinical manifestations of patients are diverse and often include abnormal uterine bleeding, unexpected elevation of β‐hCG levels, uterine enlargement disproportionate to gestational age, presence of ovarian cysts, and early onset of preeclampsia [14]. In some patients, symptoms resulting from metastasis to organs such as the lungs, liver, and brain may be the initial presentation. Diagnosis of GTD is based on a combination of clinical findings, imaging (especially ultrasound), and measurement of β‐hCG levels. In suspicious cases, histopathological examination of expelled tissue or a uterine biopsy aids in definitive diagnosis [15, 16].

One of the prominent features of this group of diseases is the possibility of early diagnosis and a favorable prognosis with appropriate management. Despite their neoplastic nature and metastatic potential, GTDs are among the few malignancies that respond very well to treatment, with patient survival rates exceeding 90% even in advanced stages [17]. However, treatment success depends entirely on early diagnosis and meticulous follow‐up. Regular and serial monitoring of β‐hCG levels plays a key role in the timely detection of recurrence and the prevention of disease progression. Because epidemiological, genetic, nutritional, and cultural patterns vary in different regions of the world, studying the clinical and biochemical characteristics of GTD in each community is of special importance. Regional studies can provide insights into risk factors and prevalence patterns that facilitate the development of preventive strategies and improvement of clinical care [18, 19].

Imam Reza Hospital in Kermanshah, as one of the referral treatment centers in the northwest of the country, annually admits a considerable number of patients with gestational trophoblastic diseases. Analyzing data from these patients can provide a clear picture of the epidemiological status, related risk factors, and clinical and biochemical indices in this region. Accordingly, the present study was designed and conducted to investigate the prevalence, associated factors, and clinical and biochemical indices of gestational trophoblastic tumors in patients referring to Imam Reza Hospital, Kermanshah, over the years 2017 to 2022.

## 2 Materials and Methods

This cross‐sectional, descriptive‐analytical study was conducted to investigate the prevalence, associated factors, and clinical and biochemical characteristics of patients with gestational trophoblastic neoplasia (GTN). The study population included all women diagnosed with GTN who were admitted and treated at Imam Reza Hospital in Kermanshah between 2017 and 2022. This hospital is a major educational and therapeutic referral center in northwestern Iran, serving as a training center for obstetrics and gynecology residents. All examinations and diagnoses were performed under the supervision of specialized obstetricians. The reporting of this study followed the recommendations of the Strengthening the Reporting of Observational Studies in Epidemiology (STROBE) Statement for cross‐sectional studies [20].

### 2.1 Sampling

A census sampling method was applied, meaning that all eligible patients during the study period were included. The inclusion criteria were women with a confirmed diagnosis of GTN, based on clinical, laboratory (β‐hCG), and/or pathological findings. Patients with incomplete medical records or missing key information were excluded from the study.

Data were extracted from both the paper‐based and electronic medical record systems of the hospital. All patient information was kept confidential and anonymized. The study was approved by the Ethics Committee of Kermanshah University of Medical Sciences (ethical code: IR.KUMS.MED.REC.1401.233), and patient privacy and confidentiality were strictly maintained. Due to the retrospective nature of the research and the use of fully anonymized patient data, obtaining individual informed consent from participants was not feasible. However, this study protocol, including the waiver of the requirement for obtaining individual informed consent, was reviewed and officially approved by the Ethics Committee of Kermanshah University of Medical Sciences. The approval was granted based on the condition that patient confidentiality and privacy are strictly maintained, which we have ensured throughout our research.

### 2.2 Data Collection Tools and Procedures

After obtaining the necessary approvals, a list of GTN patients was retrieved from the hospital's medical records department. Data were collected using a standardized checklist developed by the researcher based on a literature review and validated by ten faculty members of the Department of Obstetrics and Gynecology. The checklist included the following domains:

Demographic information: age, place of residence, educational level, occupation, and blood group.

Medical and obstetric history: number of pregnancies, history of miscarriage or hydatidiform mole, prior curettage, use of contraceptive methods, consanguineous marriage, and underlying conditions such as anemia, preeclampsia, diabetes, hypertension, and other chronic diseases.

Current pregnancy and clinical features: gestational age at diagnosis and evacuation, abnormal vaginal bleeding, uterine enlargement relative to gestational age, preeclampsia, theca‐lutein ovarian cysts, abdominal pain or distension, and other GTN‐related signs.

Therapeutic and biochemical parameters: type of intervention including uterine evacuation, chemotherapy, hysterectomy or combination therapy, initial β‐hCG levels, and metastatic status (presence and location of metastases in the lung, vagina, liver, or multiple sites).

### 2.3 Data Analysis

Collected data were entered into SPSS version 26 (IBM Corp., Armonk, NY, USA) for statistical analysis. Normality of continuous variables was assessed using skewness and kurtosis indices with their standard errors. Descriptive statistics were reported as mean ± standard deviation (SD) and median with interquartile range (IQR) for continuous variables, and as frequencies (n) and percentages (%) for categorical variables. The overall prevalence of GTN and its subtypes was calculated as the number of cases relative to the total number of live births over the 5‐year study period and presented as percentages. Additionally, the prevalence of each GTN subtype was calculated by dividing the number of cases of each type by the total number of GTN patients (n = 382) and expressed as percentages. No inferential statistical comparisons between groups were performed, as this study was descriptive in nature.

## 3 Results

A total of 382 patients were diagnosed with gestational trophoblastic disease (GTD) over the 5‐year study period. The mean age was 30.2 ± 7.01 years, with a median of 30 years (IQR: 25–35). The largest age group was 26–35 years (37.4%), followed by 19–25 years (34.8%). The mean gestational age at diagnosis was 9.6 ± 2.7 weeks, and at evacuation was 10.9 ± 2.9 weeks. Regarding gravidity, 33.2% of women were experiencing their first pregnancy, 25.9% were in their second, 15.2% in their third, and 25.7% had four or more pregnancies. More than half of the patients (52.6%) had completed only elementary school, 29.6% were illiterate, and 4.5% held a university degree. Most women were housewives (97.4%). History of miscarriage, prior molar pregnancy, and previous curettage were reported in 22.8%, 10.5%, and 25.7% of patients, respectively. Previous use of oral contraceptives was noted in 31.7% of cases. Blood group A was the most common (47.4%), followed by blood group B (25.7%), blood group O (22.3%), and blood group AB (4.7%), with 88.2% of individuals being Rh‐positive and 11.8% being Rh‐negative. Consanguineous marriage was reported in 15.4% of patients, and 30.1% had a history of chronic illness. A majority resided in urban areas (59.9%), while 40.1% lived in rural regions (Table 1).

**Table 1:** Sociodemographic characteristics of subjects (*n* = 382).

Variables		*N* (%)
**Women's age (year)**	Mean ± SD	30.2 ± 7.01
	=< 18	5 (1.3%)
	19‐25	133 (34.8%)
	26‐35	143 (37.4%)
	36‐45	98 (25.7%)
	46‐54	3 (0.8%)
**Gestational age at diagnosis (weeks)**	Mean ± SD	9.6 ± 2.7
**Gestational age at evacuation (weeks)**	Mean ± SD	10.9 ± 2.9
**Gravidity**	Gravid 1	127 (33.2%)
	Gravid 2	99 (25.9%)
	Gravid 3	58 (15.2%)
	Gravid = > 4	98 (25.7%)
**Education**	Illiterate	113 (29.6%)
	Elementary school	201 (52.6%)
	Guidance or High school	36 (9.4%)
	Diploma	15 (3.9%)
	University	17 (4.5%)
**Occupation**	Housewife	372 (97.4%)
	Administrative employee	8 (2.1%)
	Self‐employed	2 (0.5%)
**Previous miscarriages**	Yes	87 (22.8%)
	No	295 (77.2%)
**History of molar pregnancy**	Yes	40 (10.5%)
	No	342 (89.5%)
**History of curettage**	Yes	98 (25.7%)
	No	284 (74.3%)
**Previous use of oral contraceptives**	Yes	121(31.7%)
	No	261 (68.3%)
**Blood group of the mother**	AB	18 (4.7%)
	B	98 (25.7%)
	A	181 (47.4%)
	O	85 (22.3%)
**Rh factor**	Positive	337 (88.2%)
	Negative	45 (11.8%)
**Consanguineous marriage**	Yes	59 (15.4%)
	No	323 (84.6%)
**Residence**	Urban	229 (59.9%)
	Rural	153 (40.1%)
**History of chronic illness**	Yes	115 (30.1%)
	No	267 (69.9%)

During the study period, 30,133 deliveries occurred at Imam Reza Hospital, among which 382 cases of GTD were identified, yielding an overall prevalence of 1.27%. Complete mole was the most frequent type (217 cases; 56.8%; prevalence 0.72%), followed by partial mole (144 cases; 37.7%; prevalence 0.48%). Invasive mole and choriocarcinoma were less common (16 cases, 4.2%, and 5 cases, 1.3%, respectively; prevalence 0.05% and 0.02%). No cases of placental site trophoblastic tumor (PSTT) or epithelioid trophoblastic tumor (ETT) were detected (Table 2).

**Table 2:** Prevalence and distribution of gestational trophoblastic diseases (GTD) among deliveries (2017–2022, Imam Reza Hospital, Kermanshah).

Type of GTD	Number of cases (*n*)	Percentage among GTD (%)	Prevalence per deliveries (*n* = 30133)^a^
Complete mole	217	56.8	o.72
Partial mole	144	37.7	0.48
Invasive mole	16	4.2	0.05
Choriocarcinoma	5	1.3	0.02
Total	382	100	1.27

a Prevalence per deliveries = (Number of GTD cases/Total deliveries) × 100

At presentation, vaginal bleeding (85.6%) and uterine enlargement (74.6%) were the predominant clinical features. Other symptoms included abdominal pain or distension (7.6%), theca‐lutein ovarian cysts (5.8%), vomiting (5.2%), preeclampsia (2.4%), pulmonary embolism (2.1%), anemia (0.5%), and hyperthyroidism (0.3%). Multiple concurrent symptoms were observed in several patients. Serum β‐hCG levels were ≥ 100,000 mIU/mL in 61.5% of patients and < 100,000 mIU/mL in 38.5% (Table 3).

**Table 3:** Clinical presentations and serum β‐hCG levels in patients with gestational trophoblastic disease (*n* = 382).

	Frequency (*n*)	Percentage (%)
**Clinical symptoms at presentation**		
Vaginal bleeding	327	85.6%
Enlarged uterus	285	74.6%
Abdominal pain/distension	29	7.6%
Ovarian theca‐lutein cysts	22	5.8%
Vomiting	20	5.2%
Preeclampsia	9	2.4%
Pulmonary embolism	8	2.1%
Anemia	2	0.5%
Hyperthyroidism	1	0.3%
**Pre‐treatment serum β‐hCG level**		
≥ 100,000 mIU/mL	235	61.5%
< 100,000 mIU/mL	147	38.5%

Uterine evacuation (curettage) was the main therapeutic approach (85.3%). Chemotherapy alone was given to 12 patients (3.1%), and combined curettage plus chemotherapy was administered to 17 patients (4.5%). Hysterotomy and hysterectomy were performed in 7 (1.8%) and 11 patients (2.9%), respectively, while 9 patients (2.4%) underwent both curettage and hysterectomy (Table 4).

**Table 4:** Treatment modalities in patients with gestational trophoblastic disease (*n* = 382).

Treatment modality	Frequency (*n*)	Percentage (%)
Uterine evacuation (curettage)	326	85.3%
Hysterotomy	7	1.8%
Hysterectomy	11	2.9%
Chemotherapy	12	3.1%
Curettage + chemotherapy	17	4.5%
Curettage + hysterectomy	9	2.4%
Total	382	100

Metastatic disease was present in 29 patients (7.6%). The lung was the most commonly affected site (21 patients; 72.4%), followed by the vagina (8 patients; 27.6%) and liver (2 patients; 6.9%). Some patients had metastases in more than one site. These findings underscore the lung as the predominant site of GTD dissemination (Table 5).

**Table 5:** Metastatic status and sites of metastasis in patients with gestational trophoblastic disease (*n* = 382).

Variables	Frequency (*n*)	Percentage (%)
**Metastasis**		
Present	29	7.6%
Absent	353	92.4%
**Sites of metastasis** a		
Lung	21	72.4%
Vagina	8	27.6%
Liver	2	6.9%

a Some patients had multiple metastatic sites, so the total percentage may exceed 100%

## 4 Discussion

This study aimed to investigate the prevalence, associated factors, and clinical and biochemical indices of patients with gestational trophoblastic neoplasia (GTN) at Imam Reza Hospital in Kermanshah. The results showed that the overall prevalence of GTN in this center was 1.27% of all deliveries, indicating a significant burden of this disease in the studied population. Complete mole was the most common type, accounting for 56.8%, and the predominant symptoms included vaginal bleeding and uterine enlargement, observed in more than 85% and 74% of patients, respectively. These findings align with international reports, which identify complete mole as the most frequent form of gestational trophoblastic disease and vaginal bleeding as the primary clinical symptom. Demographically, most patients were in the active reproductive age group (26–35 years), and over half had low education levels or were illiterate, which may affect access to healthcare services and disease awareness. Given that GTN primarily affects women of reproductive age, fertility preservation represents an important consideration in clinical management, particularly as cancer survivorship trends increasingly emphasize quality‐of‐life outcomes including future fertility [21]. A history of molar pregnancy was reported in 10.5% of patients, confirming the importance of this risk factor in the recurrence of GTN.

The results of the present study regarding prevalence and clinical patterns are comparable with both international and domestic reports. For example, the national study by Yamamoto et al. in Japan [22] reported a hydatidiform mole prevalence of 2.02 per 1000 live births, and low‐risk and high‐risk GTN incidences of 15.3 and 3.5 per 100,000 live births, respectively. These rates are lower than those observed in our study, which could be attributed to demographic differences, healthcare access, and the existence of a national registry system in Japan. In Iran, several studies have been conducted. Mohammadjafari et al. [23] reported a mole prevalence of 54 per 1000 pregnancies in Ahvaz, which is considerably higher than our findings; they also found a significant association between blood groups A and O and complete mole. In Karimi‐Zarchi et al.‘s study in Yazd [24], complete mole (54.6%) was the most common pathology, and vaginal bleeding (90%) was the main symptom, findings consistent with our study. They also reported a history of molar pregnancy (4%) and positive family history (9.4%) as risk factors, highlighting the importance of investigating genetic aspects. Differences in prevalence and patient characteristics likely result from geographic heterogeneity, diagnostic methods, registry criteria, and socio‐economic and genetic variations. Generally, the consistency in clinical symptoms (vaginal bleeding and uterine enlargement) suggests these are sensitive primary indicators for screening and early diagnosis of GTN.

Metastasis evaluation in this study showed that 7.6% of patients had metastatic involvement, with the lung being the most common site (72.4%), followed by the vagina (27.6%) and liver (6.9%). Understanding this pattern has clinical significance as it guides the therapeutic approach and intervention timing. All metastatic patients received systemic chemotherapy, with surgery required in some cases [25, 26]. This aligns with findings by Yamamoto et al., who identified lungs as the prevalent metastasis site in high‐risk GTN patients [22]. In cases where cross‐sectional imaging is required for metastasis workup, the use of contrast agents during pregnancy requires careful risk‐benefit assessment, particularly given the potential teratogenic concerns in early gestation [27].

The study demonstrated that vaginal bleeding and uterine enlargement are sensitive early diagnostic indicators for GTN. Demographically, reproductive age and low educational levels played important roles in disease occurrence, underlining the need for educational programs and awareness raising in high‐risk groups. These findings underscore the influence of socio‐economic factors and literacy on healthcare access and symptom recognition. Our results are consistent with another study showing that age, pregnancy history, β‐hCG levels, and parity are significant factors associated with GTN, while the interval since last pregnancy was not statistically significant [1]. Furthermore, another study on 120 GTD patients reported vaginal bleeding as the most common complaint (85.8%) and showed moderate agreement between initial clinical diagnosis and definitive pathology (Kappa=0.419, *p* < 0.001), emphasizing the limitations of solely clinical diagnosis and the necessity of confirmatory pathological and biochemical tests [28].

Blood group distribution in our study revealed that blood type A was the most frequent, aligning with recent findings from Bangladesh, where 48.6% of GTN patients had blood group A [29]. However, in the absence of regional population‐based blood group data for the general healthy population, no definitive inference can be made regarding blood group as a risk factor for GTN. Additionally, 15.4% of patients in our study reported consanguineous marriages, stressing the importance of investigating genetic and familial factors in future research [30].

### 4.1 Limitation of Study

Limitations include the cross‐sectional design, which does not allow causal inferences; data from a single center limiting generalizability; the absence of detailed genetic and socio‐economic data; a lack of information on treatment outcomes; and the unavailability of regional population‐based blood group data for the general healthy population, which precluded comparison of blood group frequencies between GTN patients and the healthy general population. Long‐term follow‐up data were unavailable, preventing full assessment of late complications, recurrence, and survival. Diagnostic challenges, especially regarding clinical and pathological heterogeneity of rare variants like mixed trophoblastic tumors, require thorough pathological and genetic evaluation.

### 4.2 Strength of Study

Strengths of this study include a comprehensive sampling over 5 years and the use of standardized checklists, ensuring data accuracy and consistency. Appropriate statistical analyses and the use of precise clinical and biochemical indices are other advantages.

### 4.3 Suggestions

Considering reports of rare and mixed GTN types, future studies should explore molecular aspects and novel therapies such as immunotherapy for resistant cases. Additionally, given the reproductive age of most GTN patients in our cohort, investigation of fertility preservation strategies and long‐term obstetric outcomes represents an important direction for future research. Overall, these limitations suggest that the establishment of a coherent registry system for trophoblastic diseases is essential to accurately monitor prevalence, risk factors, outcomes, and improve the quality of care.

## 5 Conclusion

The significant prevalence of gestational trophoblastic neoplastic in the study population, particularly the high rate of complete mole, highlights the importance of this disease as a prominent health issue in the region. Reproductive age and low education levels are key factors associated with disease occurrence, emphasizing the need for education and awareness improvement in high‐risk groups. A history of molar pregnancy was confirmed as the most important risk factor for disease recurrence, and post‐treatment follow‐up should focus on this patient group. The treatments applied rely on the appropriate use of uterine evacuation, chemotherapy, and surgery according to established clinical protocols. This study can play a crucial role in improving strategies for the prevention, diagnosis, and treatment of GTN in similar regions and motivate future research on genetic and social factors.

## Author Contributions

**Nasrin Mansouri:** conceptualization, investigation, funding acquisition, writing – original draft, writing – review and editing, supervision, project administration. **Ali Azizi:** conceptualization, writing – review and editing, supervision, data curation, formal analysis. **Samira Sajadi Asl:** conceptualization, formal analysis, software, data curation, methodology, writing – original draft, writing – review and editing. **Zahra Javanbakht:** conceptualization, writing – review and editing, writing – original draft, project administration, methodology. **Marzieh Bagherinia:** conceptualization, investigation, funding acquisition, writing – original draft, resources, formal analysis, writing – review and editing.

## Funding

The authors have nothing to report.

## Ethics Statement

The study was conducted by the Declaration of Helsinki, and the study was approved by Kermanshah University of Medical Sciences, Kermanshah, Iran, with the ethical code IR. KUMS. MED. REC.1401.233.

## Consent

The authors have nothing to report.

## Conflicts of Interest

The authors declare no conflicts of interest.

## 1 Transparency Statement

All authors have read and approved the final version of the manuscript. The lead author, Marzieh Bagherinia, certifies that this manuscript is an honest, accurate, and transparent account of the study reported; that no important aspects of the study have been omitted; and that any discrepancies from the study as planned (and registered, if applicable) have been reported. from the study as planned (and, if relevant, reported) have been explained. The corresponding author had full access to all study data and takes complete responsibility for the integrity and accuracy of the data analysis.

## Supporting information

Supporting File 1

Supporting File 2

## Data Availability

The data that support the findings of this study are available from the corresponding author upon reasonable request.
